# Analysis of the canid Y-chromosome phylogeny using short-read sequencing data reveals the presence of distinct haplogroups among Neolithic European dogs

**DOI:** 10.1186/s12864-018-4749-z

**Published:** 2018-05-10

**Authors:** Matthew T. Oetjens, Axel Martin, Krishna R. Veeramah, Jeffrey M. Kidd

**Affiliations:** 1 Department of Human Genetics, University of Michigan Medical School, Ann Arbor, MI USA; 2 Department of Ecology and Evolution, Stony Brook University, Stony Brook, New York, USA; 3 Department of Computational Medicine & Bioinformatics, University of Michigan Medical School, Ann Arbor, MI USA

**Keywords:** Canid, Y-chromosome haplogroups, Ancient dog

## Abstract

**Background:**

Most genetic analyses of ancient and modern dogs have focused on variation in the autosomes or on the mitochondria. Mitochondrial DNA is more easily obtained from ancient samples than nuclear DNA and mitochondrial analyses have revealed important insights into the evolutionary history of canids. Utilizing a recently published dog Y-chromosome reference, we analyzed Y-chromosome sequence across a diverse collection of canids and determined the Y haplogroup of three ancient European dogs.

**Results:**

We identified 1121 biallelic Y-chromosome SNVs using whole-genome sequences from 118 canids and defined variants diagnostic to distinct dog Y haplogroups. Similar to that of the mitochondria and previous more limited studies of Y diversity, we observe several deep splits in the Y-chromosome tree which may be the result of retained Y-chromosome diversity which predates dog domestication or post-domestication admixture with wolves. We find that Y-chromosomes from three ancient European dogs (4700–7000 years old) belong to distinct clades.

**Conclusions:**

We estimate that the time to the most recent comment ancestor of dog Y haplogroups is 68–151 thousand years ago. Analysis of three Y-chromosomes from the Neolithic confirms long stranding population structure among European dogs.

**Electronic supplementary material:**

The online version of this article (10.1186/s12864-018-4749-z) contains supplementary material, which is available to authorized users.

## Background

Dogs are a domesticated canid lineage likely descended from a now-extinct population of Eurasian grey wolves [1]. Multiple approaches have been used to explore the genetic history and diversity of dogs, a question which is complicated by long term population structure and both ancient and modern gene flow among dogs and wolves [2]. The different genomic compartments which have unique patterns of inheritance (i.e. autosomes, mitochondria, and the sex chromosomes) have each revealed novel, and sometimes conflicting, aspects of dog evolution [3–7]. The Y-chromosome and the mitochondria are present as single, largely non-recombining haplotypes and each thus represents a single locus sampled from the evolutionary history of a species. Due to their inheritance patterns, the Y-chromosome and the mitochondria have a reduced effective population size and are more sensitive to the impact of genetic drift. Nonetheless, the single trees represented by these uniparentally inherited loci can be informative for reconstructing population history [8]. Due to their increased copy number per cell, mitochondrial DNA is more easily obtained from ancient samples than nuclear DNA. Thus, mitochondrial analysis has been a key feature of studies involving ancient DNA and have revealed important insights into the evolutionary history of canids [6, 9].

Canid mitochondrial phylogenies show that dogs and wolves are not reciprocally monophyletic [6]. The mitochondrial tree contains four deeply rooted clades encompassing dogs and many grey wolf groups. These four clades form the basis of dog mitochondrial haplogroup assignment, known as haplogroups A-D [6, 10]. The time of the most recent common ancestor (TMRCA) of haplogroups A-D significantly predates estimates for domestication based on archeological and genetic evidence [1, 4, 6, 9, 11, 12]. Instead, these clades may represent variation present among the founding population of the dog lineage or the results of wolf introgressions into dog populations. The relative frequencies of mitochondria haplogroups are not stable over time, with changes reflecting processes such as drift, migration, and population growth. Although the mitochondria A and B haplogroups are most common in contemporary European dogs, surveys of ancient samples indicate that the majority of ancient European dogs carried the C or D mitochondrial haplotype. This apparent turnover in mitochondrial haplogroups may reflect the migration of a distinct dog population into Europe over the past 15,000 years [9].

Relative to the mitochondria, comparatively little is known about the Y-chromosome haplogroup diversity present among ancient dogs in Europe. Similar to the mitochondria, studies of contemporary samples show that the Y-chromosome tree is characterized by deep splits among dogs [7]. A study of Y-chromosome sequence based on a partial assembly (14.4 kb in length) collected from 151 dogs revealed five haplogroups [13]. Larger studies of hundreds of samples using genotyping arrays designed to include Y-chromosome markers show a high diversity of Y-chromosome haplotypes across Africa, India, Central Asia and Southwest Asia [3].

Hundreds of dog and wolf samples have been sequenced using short-read technology, but there has been limited focus on dog Y-chromosome evolution. In part, this is because the presence of highly duplicated sequences, known as amplicons necessitates special care in variant calling. Recently, a canine Y-chromosome reference was sequenced using 454-sequencing applied to pools of BAC clones [14]. This sequence includes a ~ 1 Mb region of amplicon sequence which includes multiple copies of *SRY*, duplication of which has also been observed in pigs and rabbits [15, 16]. Here, we utilize this Y-chromosome reference along with whole genome sequence data from 118 samples to determine the Y-chromosome haplotype diversity found among contemporary and ancient dogs. First, we utilize publically available short-read data to identify regions of the Y-chromosome amendable to variant identification. Using this map of callable regions, we resolve the Y-chromosome phylogeny and define SNVs diagnostic to each haplogroup using publically available genome sequence data from 118 samples. We then assess the geographic distribution of each haplogroup among modern dogs based on autosomal ancestry inferred using genome sequence data from 104 male dogs. Finally, we interrogate the previously published sequences of three ancient dog genomes to provide an initial assessment of Y-chromosome diversity in Europe during the Neolithic.

## Results

### Identifying Y-chromosome segments suitable for SNV calling

**Fig. 1:**
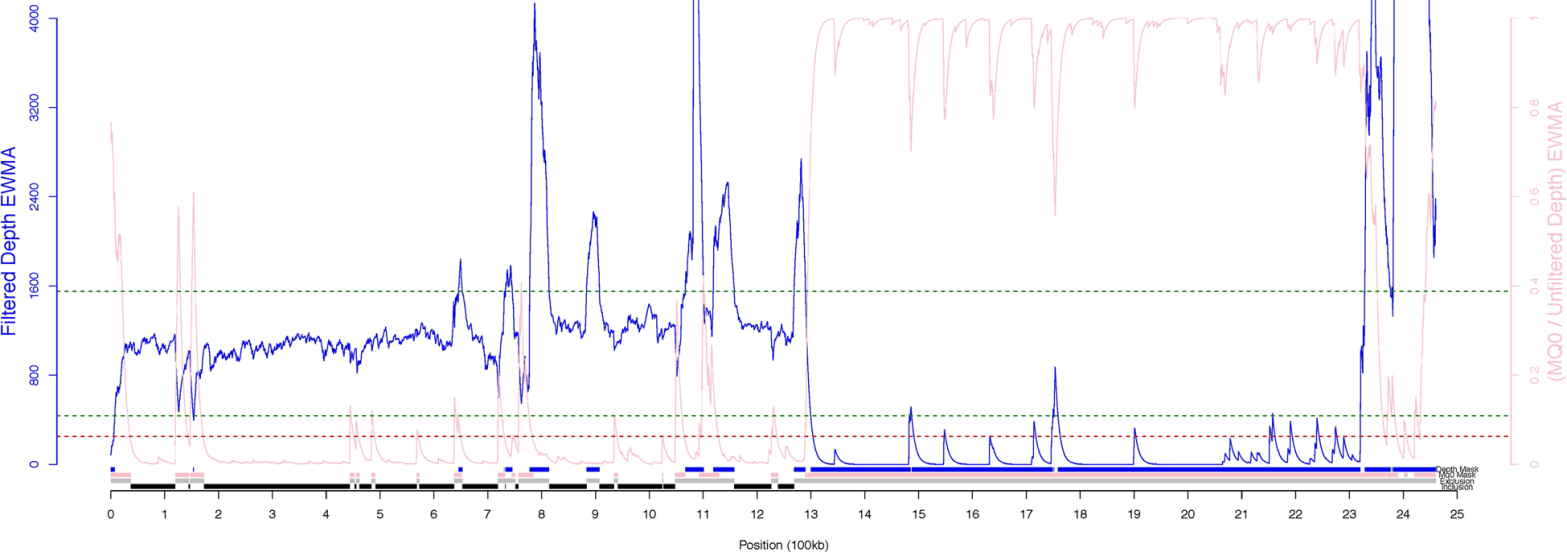
Regional callability mask to identify regions suitable for variant calling Exponentially weighted moving averages (EWMA) of read depth (blue line) and the mq0/unfiltered depth ratio (pink line) are plotted along the Y-chromosome sequence (KP081776.1). The dashed lines represent maximum and minimum thresholds for filtered depth (green) and a maximum threshold for the mq0 ratio (red). Colored bars below the plot indicate regions masked by the depth filter (blue), masked by the mq0 ratio filter (pink), excluded from the analysis (grey), and included (black) prior to site-level filtering

We mapped Illumina short read libraries from a diverse collection of 118 publically available canid genomes to the Li et al. canine Y-chromosome assembly (Additional file 1: Table S1). This sample set includes 1 coyote [17], 13 wolves [4, 17–20], 30 village dogs including samples from India, Portugal, Nigeria, and China [4, 21, 22], and 74 breed dogs [4, 19–25]. Based on metrics such as raw read depth, MQ0-to-depth ratio, and the apparent presence of heterozygous variant calls, we identified 484,924 positions on the Y assembly amendable to variant identification using short-sequencing reads (Fig. 1, Additional file 2: Table S2) [26, 27]. As expected, the regions that pass this callability mask are located outside of the boundaries of amplicons identified by a dotplot analysis of the existing assembly (Additional file 3: Figure S1, Additional file 4: Table S3). Since amplicons may be dynamic over evolutionary time, we also constructed a callability mask using reads only from the coyote sample (Additional file 5: Figure S2). The coyote mask is nearly identical to that obtained from the combined canid dataset, suggesting that divergence between coyote and dog will not lead to large-scale errors in read mapping for this analysis. In total, we identified 1221 biallelic Y-chromosome SNVs across the 118 analyzed samples.

### Canid Y-chromosome phylogeny

**Fig. 2:**
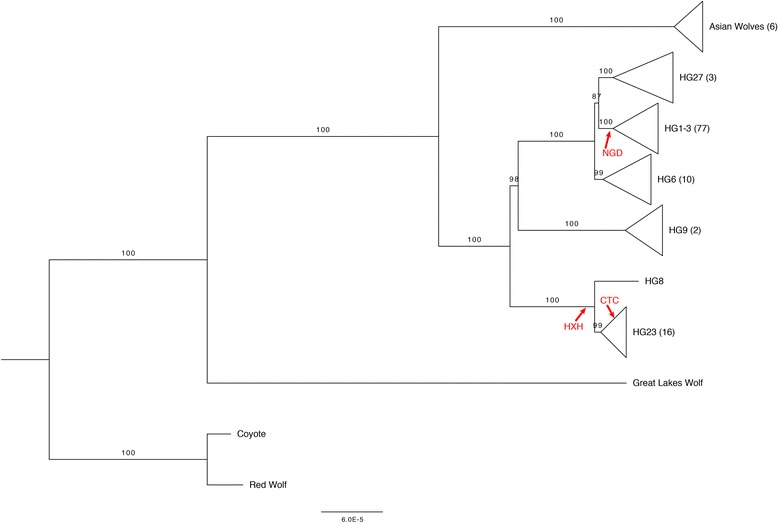
Maximum likelihood phylogeny of 118 candid Y-chromosomes A Y-chromosome haplogroup tree produced by RAxML (8.1.13) using the GTR+ I model is depicted. Clades in the tree have been collapsed by haplogroup assignment. The number of samples within each collapsed node is indicated in parentheses next to the haplogroup assignment. For each node, percent bootstrap support out of 1000 iterations is indicated above the branch. The locations of three ancient samples, based on the presence of diagnostic mutations, are indicated in red

To assign haplogroup labels and aid comparisons with prior studies, we remapped 151 markers present on the 170 K Illumina HD Canine SNP Array to the Li et al. Y-chromosome assembly [3]. This included seven derived alleles specific to the HG8 haplogroup, eight specific to the HG6 haplogroup, and two specific to the HG23 haplogroup. We reclassified some haplotypes, and refer to the four HG1–3 haplotypes Hb.1, H27, H5b and H5a collectively as HG27. The 170 K Illumina HD Canine SNP Array contains a single diagnostic site specific to the HG27 haplogroup as well as one site specific to HG1–3. Additionally, a variant diagnostic for the HG9 haplogroup [7] was remapped to the Li et al. reference and included in the present analysis. Using genotypes at these sites, we assigned 109 Y-chromosomes to one of these six major dog haplogroups. Most of the Y-chromosomes in our dataset belong to the HG1–3 clade (*n* = 77), followed by HG23 (*n* = 16), HG6 (*n* = 10), HG27 (*n* = 3), HG9 (*n* = 2), and HG8 (n = 1). Each haplogroup is represented by distinct nodes in maximum likelihood phylogeny constructed from the full set of 1221 SNVs discovered in the resequencing data (Fig. 2). The structure of the phylogeny reveals that HG1–3, HG27 and their sister clade HG6 share a common ancestor that emerged long after their split from HG9. Haplogroup HG8, predominantly found in Africa and the Middle East, is represented by one of the two Nigerian village dogs included in this study, and is related to broadly distributed HG23 haplogroup [3].

Five gray wolf samples were carriers of dog haplogroups (HG1–3 clade (*n* = 1); HG23 (*n* = 2); HG6 (n = 1); HG9 (n = 1)) and are often represented as deep branches within their respective clades (Additional file 6: Figure S3). Recent gene flow or admixture is one potential explanation for the presence of dog haplogroups in these wolves. We performed a model based ancestry analysis of the included dogs and wolves based on autosomal genetic variation discovered from the whole genome sequencing data. This analysis did not identify a substantial degree of dog ancestry in these wolf samples (Additional file 7: Figure S4); however, the Nigerian village dog NG03, which is assigned to the HG9 haplogroup along with a Chinese wolf sample, has detectable wolf ancestry (Additional file 7: Figure S4, K = 3). Similar levels of wolf ancestry were also detected in other village dogs from Nigeria, India, and Portugal.

Additionally, we observed a distinct wolf patriline found in six samples from the Shanxi, Qinghai, Xinjiang, and Tibetan regions. This branch, which we refer to here as Asian Wolves, is comparably deep with respect to the tree and is consistent with a split prior to the formation of the dog haplogroups. However, we note that other Asian wolves carry Y-chromosomes which cluster with the dog HG9 (a Xinjiang wolf) or HG23 haplogroups (a Xinjiang and an Indian wolf).

The red wolf-coyote clade and the Great Lakes wolf represent the deepest branches in the canid phylogeny. Consistent with previous analyses of canid Y-chromosomes and mitochondria [6, 7], the coyote Y-chromosome is divergent from the grey wolf-dog clade. Instead, the coyote shares a clade with the red wolf, a group known to contain a high amount of coyote admixture [28, 29]. The single male Great Lakes wolf (a sample from Minnesota [19] carries a strikingly divergent Y-chromosome, with 199 derived alleles (16% of the total SNVs) unique to this Y-chromosome.

To infer the TMRCA of Y-chromosome clades, we used the Bayesian Markov chain Monte Carlo approach implemented in BEAST, which yielded the same tree topology as obtained with RAxML (Fig. 2). We calibrated our estimates by setting a prior TMRCA at the root of the phylogeny as 1.5 million years ago (mya) based on a previous estimate of the dog/wolf-coyote divergence time [30, 31]. Based on relaxed and strict molecular clock models, we find that haplogroup TMRCAs range from ~ 70,000 years (HG23) to ~ 159,000 years (HG6) (Table 1). On average, we find 362.81 (s.d. = 8.63) substitutions between dog/wolves and coyote. From this estimate, a naïve counting approach yields a Y-chromosome substitution rate at 2.49 × 10^− 10^ substitutions per site per year, a value slightly smaller than that obtained from a strict (2.86 × 10^− 10^ substitutions per site per year, 95% HPD: 2.00–3.679 × 10^− 10^) or relaxed molecular clock model (3.07 × 10^− 10^ substitutions per site per year, 95% HPD: 1.24–5.15 × 10^− 10^).

**Table 1:** TMRCA Values

Branch	Diagnostic Mutations	TMRCA ^a^ (Relaxed Clock)	ESS ^b^	TMRCA ^a^ (Strict Clock)	ESS ^b^
HG1–3; HG27; HG6; HG9; HG8; HG23; AW; GLW;	150	1.4938 [0.6896, 2.6717]	1740	1.459 [1.0653, 1.8996]	11,096
HG1–3; HG27; HG6; HG9; HG8; HG23; AW;	109	0.7676 [0.3031, 1.3928]	1139	0.77 [0.5463, 1.0179]	3436
HG1–3; HG27; HG6; HG9; HG8; HG23;	34	0.4772 [0.1917, 0.8899]	934	0.4792 [0.3336, 0.6367]	5806
HG1–3; HG27; HG6; HG9;	4	0.4373 [0.173, 0.8129]	930	0.446 [0.3136, 0.5989]	5763
HG1–3; HG27; HG6;	33	0.2019 [0.0803, 0.3651]	1021	0.1948 [0.1332, 0.2631]	4671
HG1–3; HG27;	2	0.1805 [0.073, 0.3285]	1024	0.1765 [0.1201, 0.239]	5466
HG8; HG23;	40	0.1139 [0.0387, 0.2173]	1081	0.1172 [0.0702, 0.1675]	6907
HG1–3	7	0.0975 [0.0366, 0.1811]	1113	0.099 [0.0614, 0.1395]	5466
HG27	7	0.138 [0.0525, 0.2543]	1023	0.1387 [0.0917, 0.1908]	5359
HG6	4	0.1599 [0.0642, 0.2962]	1090	0.1578 [0.1045, 0.2171]	5807
HG9	50	0.0923 [0.0258, 0.184]	1138	0.0907 [0.0509, 0.1359]	7075
HG23	2	0.0715 [0.0246, 0.1352]	1027	0.0708 [0.0418, 0.1045]	3861
HG8	20	NA	NA	NA	
Asian Wolves	112	0.0636 [0.0202, 0.1240]	919	0.0562 [0.0308, 0.0838]	3646
Great Lakes Wolf	199	NA	NA	NA	
Incompatible	21	NA	NA	NA	

^a^TMRCA in millions of years, with 95% highest posterior density interval

^b^Estimated effective sample size

### Autosomal genetic ancestry and Y-chromosome Haplogroups

**Fig. 3:**
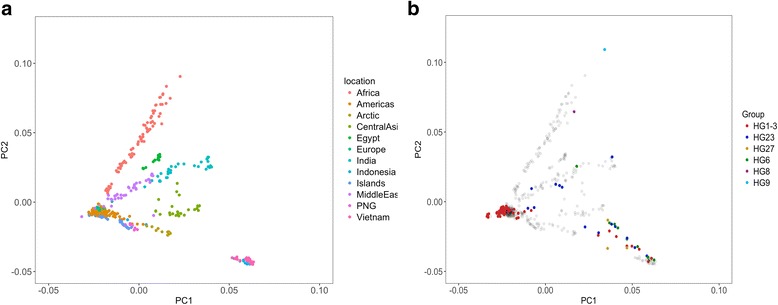
The relationship between autosomal ancestry and Y-chromosome haplogroups Major groupings of canine ancestry are shown based on a principal components analysis of autosomal markers from 499 village dogs from Shannon et al. **a**. The geographic origin of each sample is indicated by color. The 104 male dogs used in this study are projected onto the resulting principal components and colored based on haplogroup (**b**). Village dogs from (**a**) are shown as transparent dots in (**b**)

Next, we assessed the relationship between Y-chromosome haplogroups and genetic ancestry using principal components analysis. We defined ancestry based on variation at the autosomal positons present on the 170 K Illumina HD Canine SNP Array. First, we defined broad patterns of geographic diversity based on 499 village dogs (Fig. 3a) which have known ancestry and have been previously genotyped at these positions by Shannon et al. [3]. Next, we used the whole genome sequencing data to genotype the 104 male dogs used in this study at these autosomal positions. The resulting genotypes were then projected onto the PC space defined by the 499 village dogs (Fig. 3b). Based on this assessment of autosomal ancestry, we confirm that the breed dogs carrying the HG1–3 haplogroup mostly cluster with village dogs from Europe and the Americas with some exceptions: a Husky with the HG1–3 haplogroup clustered with the Arctic samples and six HG1–3 Chinese Village dogs from the Diqing, Lijiang, and Yingjiang regions, displayed Asian ancestry. In contrast, the HG23 haplogroup is found in samples with a broader distribution of ancestry, including village dogs from China that show the expected Asian autosomal ancestry, an Indian Village dog, as well breed dogs with Middle Eastern/Indian ancestry (Afghan Hound, Saluki, and Sloughi) as well as Tibetan Terriers whose ancestry projects near European/American center. The HG6 samples in our study include an Indian village dog, which clusters with Indian reference samples as well as Tibetan Mastiffs and Chinese village dogs that display Asian ancestry. The three HG27 samples (a Korean Jindo, Tibetan Mastiff, and Shiba Inu) have ancestry which appears intermediate between village dogs from Central Asia and Vietnam. As previously mentioned, the HG8 and HG9 haplogroups are each found in a single village dog from Nigeria, each of which projects along the variation found in Africa.

### Y-chromosome variation in three ancient dogs

To offer an initial depiction of Y-chromosome diversity among ancient dogs, we determined Y-chromosome haplotypes for three recently published ancient samples from Europe [9, 11]. This includes an ancient dog dated to ~ 4800 calendar years before the present found in the Newgrange grave complex in Ireland (abbreviated NGD), a sample from the early Neolithic site of Herxheim, Germany, dated to ~ 7000 years ago (abbreviated HXH), and a dog from the Cherry Tree Cave in Bavaria, Germany estimated to be ~ 4700 years old (abbreviated CTC). All three ancient samples were previously reported as being male. We assigned Y haplogroups using the diagnostic alleles we identified (Additional file 6: Figure S3). CTC and HXH both had the derived alleles at all the HG8-HG23 diagnostic sites (21 sites passing quality filters in both samples). Unfortunately, sites specifically diagnostic for the HG23 or HG8 clades were not callable in these two samples. However, CTC carried 2 of 4 callable derived alleles that were unique to the India wolf leaf, a HG23 haplotype. The NGD Y-chromosome belongs to the HG1–3 haplogroup (7 of 7 callable derived alleles). Unlike CTC, it did not carry any diagnostic alleles that matched any contemporary modern dogs or wolves within the HG1–3 haplogroup.

## Discussion

In this study we present an analysis of Y-chromosome sequence diversity based on mapping whole genome sequence data from 118 canids to a published canine Y-chromosome assembly. Despite the incomplete representation of Y-chromosome amplicons, the published reference provides a critical resource for phylogenetic analysis of Y-chromosome sequence variation. Our data supports the Y-chromosome haplogroup definitions previously reported [3, 7]. Relative to the Y-chromosome network presented by Shannon et al., we find that HG27 is divergent from HG1–3 and we suggest that it should should be considered a unique haplogroup.

Using the variation discovered from sequence data, we applied a Bayesian MCMC approach to estimate TMRCAs for each haplotype group. Our estimated Y-chromosome mutation rate (3.07 × 10^− 10^ substitutions per site per year, relaxed clock model) falls within the range of a previous estimate by Ding et al. who used a similar calibration and estimate 1.35 × 10^− 10^– 4.31 × 10^− 10^ substitutions per site per year [7]. The TMRCAs we estimated are substantially older than mitochondria phylogenies calibrated with tip dates of ancient samples, [6] which report clade-specific TMRCAs < 25,000 years ago. We note that our Y-chromosome TMRCA estimates are extremely sensitive to our assumptions about the age of the root of the tree and should be interpreted with caution due to the uncertainty in this single calibration point. However, the relative ages of the branches and the chronological order of haplogroup divergences are more robust than the absolute estimated dates.

In general, the relationships between Y-chromosome haplogroups and autosomal ancestry we report are very similar to the relationships described in Shannon et al. [3] As noted earlier, our dataset includes a subset of wolves with Y-chromosomes assigned to a dog Y-haplogroup. However, ADMIXTURE analysis does not indicate substantial recent dog ancestry in these samples, suggesting that their placement on the Y-chromosome phylogeny reflects variation in Y-chromosome haplotypes that was present in the ancestral population and therefore predates the domestication process or is the result of ancient introgression events whose signature of autosomal ancestry has been diluted.

The high divergence of the Y-chromosome from a single a Great Lakes wolf is unexpected. The Great Lakes wolves are hypothesized to be a long-standing ecotype of the grey wolf that persisted in the presence of genetic introgression from coyote and other grey wolves [29, 32]. A previous study of Y-chromosome microsatellite data from Great Lakes wolves did not find evidence of a unique grouping, instead finding that some Great Lakes wolves carried Y-chromosomes that clustered with coyotes while others carried haplogroups that clustered with other grey wolves [32]. The retention of such a deeply diverged lineage may be the result of strong population structure in the past history of Great Lakes wolves. Further interpretation will require a more diverse collection of Y-chromosome sequences from additional coyotes and wolves.

Using our expanded set of clade-specific mutations, we assessed the Y-chromosome haplogroups found in three recently published ancient canine genomes from Europe. The two samples from Germany, CTC and HXH, belonged to the HG8-HG23 clade which is common in contemporary dogs from Asia and the Middle East. Previously published analysis of the autosomal genome of CTC indicated shared ancestry between CTC and modern wolves that are now found in India and Iran. Consistent with this proposed ancestry, our analysis shows that the CTC Y-chromosome shares derived alleles with an Indian wolf [11]. In contrast, NGD, a sample from Ireland and a near contemporary of CTC, belongs to the HG1–3 haplogroup which is mostly found in modern European dogs. This Y-chromosome data, which shows that at least two Y-chromosome haplogroups were present among European dogs during the Neolithic, supports the existence of long-lasting population structure among European dogs.

## Conclusions

Using sequencing data, we find that the estimated TMRCA of dog Y haplogroups predates dog domestication. We further reveal the placement of several wolf Y-chromosomes within deep branches of dog haplogroup clades. Using an expanded set of mutations diagnostic for each haplogroup, we find that distinct Y haplogroups were present in Europe during the Neolithic and that CTC, a ~ 4700 year old ancient dog from Germany has a Y-chromosome that shares diagnostic alleles with wolves found in India.

## Methods

### Sample selection and Y-chromosome data processing

The canid short-read sequencing data used in this analysis are available from the NCBI Sequence Read Archive (SRA; Additional file 1: Table S1). Sequencing runs from each experiment were independently aligned to a canine reference genome that included both the CanFam3.1 sequence and the canine Y-chromosome assembly from Li. Et al. (KP081776.1) [14]. We note that KP081776.1 is present in the opposite chromosome orientation from that typically employed. Read alignment was performed with bwa mem (version 0.7.13) using -t 4 -M flags [33]. Sample identity across multiple sequencing runs was confirmed by assessment of identity-by-descent at SNPs included on the 170 K Illumina HD Canine SNP array. IBD across all runs was calculated using the Plink --genome function [34]. We removed samples with sequencing runs (> 100,000 reads) that displayed a low level of relatedness across runs (pi-hat < 0.90) to avoid merging runs derived from different biological samples. Once runs within each sample were merged, we sorted and marked duplicates of the alignment files using Picard (version 2.3.0). Base quality values in BAM files were recalibrated and variants were then calculated using the GATK haplotype caller (version 3.5–0) [35].

To identify male samples for inclusion in our study population, we calculated each sample’s average autosome to X-chromosome (A/X) sequencing depth. We included samples that had an A/X sequencing depth ratio > 1.85 and an autosomal coverage > 10×. The final sample set contained 118 samples including 1 coyote [17], 13 wolves [4, 17–20], 30 village dogs including samples from India, Portugal, Nigeria, and China [4, 21, 22], and 74 breed dogs [4, 19–25]. We note that this set includes two Afghan Hounds which are clones of each other and therefore have identical Y-chromosome sequences [25]. We next recalled Y-chromosome genotypes in the male samples using the GATK haplotype caller using the EMIT_ALL_SITES flag. Following methods previously used for identifying repetitive sequence unsuitable for read mapping in primate Y-chromosomes, we used mapping and depth statistics from the VCF info field to identify callable regions on the canine Y-chromosome (Fig. 1) [26, 27]. Once a callable region was identified, we applied site level filtering to the remaining sequence: dropping maximum likelihood heterozygotes, missing sites, and positions with an MQ0/raw depth ratio > 0.10. A second depth filter was then applied to remove positions with extreme sequencing depths (median depth ± 3 M.A.D.). We also removed positions that were within 5 bp of GATK called indels.

Three ancient canine samples previously identified as male (NGD, CTC, and HXH dogs) were aligned to our custom canine reference using the same procedure as described previously [9, 11]. In short, the damage patterns identified with mapDamage were adjusted into the confidence of the variant calling [36, 37]. We limited our analysis of ancient samples to positions with a minimum read depth of 4; genotype quality score of 30; map quality score of 15; and base quality score of 15.

### Haplogroup assignment

We assigned Y-chromosome haplogroups using the definitions from Shannon et al. based on the 207 Y-chromosome polymorphic variants captured on the 170 K Illumina HD Canine SNP Array [3]. Since the 170 K Illumina HD Canine SNP Array Y-chromosome probes were designed on a less complete Y-chromosome assembly (chrY_nonPAB), we remapped the SNP locations to the Li et al. assembly [14]. First, we extracted +/− 50 bp of sequence from each SNP position on the chrY_nonPAB assembly and determined the location of each 101 bp long fragment on the Li et al. Y-chromosome assembly using blat [38]. We filtered sequences that mapped to multiple regions at an identity > 0.99, had ambiguous orientations, or did not pass the filtering criteria mentioned above.

### Phylogenetic analysis

Maximum likelihood (ML) trees were reconstructed from the Y-chromosome sequence using RAxML (8.1.13) with 1000 bootstrap replicates [39]. The GTR+ I model was identified by jModelTest2 as the best fitting substitution model of 12 candidates as determined by the lowest AIC [40]. We estimated the time of the most recent common ancestor (TMRCA) for branches of interest using the MCMC method BEAST using both a strict and relaxed molecular clock with a log-normal prior distribution on the dog-coyote TMRCA [41, 42]. We merged two independent BEAST runs performed for 10,000,000 iterations with sampling every 1000 iterations. Convergence of MCMC chains was assessed with Tracer and analysis of consensus plotted with FigTree yielded the same tree topology as found with RAxML [42].

We assigned SNVs in our call set to branches within this phylogeny using the ETE toolkit in python [43]. For a given SNV, we identify all leaves in the tree carrying the genotype and iteratively checked deeper nodes to test if all samples are also carriers. This continued with recursion until the deepest node of genotype universality was identified. If a single top node is identified for a variant, we consider that variant diagnostic for the given node. If multiple top nodes are identified, the variant is called as potentially recurrent and is incompatible with the phylogenetic tree.

### Ancestry and geography

Autosomal genetic ancestry was visualized via a principal components analysis (PCA) using the smartPCA program from eigensoft version 3.0 [44]. For the PCA, we limited variants to those present on the 170 K Illumina HD Canine SNP Array so that the larger collection of samples from Shannon et al. [3] could be included. Here, ancestry was first estimated using the 499 village dogs from Shannon et al. [3] as this sample set more completely represents global dog genetic diversity than collections of breed dogs, and the male dogs (*n* = 104) from our study were subsequently projected onto the resulting components. The proportion of wolf and dog admixture for each dog or wolf sample was estimated using the program ADMIXTURE [45]. As input, we used autosomal genotypes from the whole genome short-read data called by GATK HaplotypeCaller. Variant quality score recalibration (VQSR) was applied to the candidate callset using the Illumina 170 K Illumina HD Canine SNP Array as training and retaining only those SNPs that pass the 99.0 tranche. The VCF file was reformatted into a binary plink file, to which we applied quality control and LD pruning. We removed individual samples (*n* = 30) selected at random from related pairs (pi_hat > 0.125). Variants were filtered with a minor allele frequency < 0.01 and/or call rate < 0.99. We pruned variants in strong LD by using the indep-pairwise command in plink with the following parameters: 50 kb window size, 10 variant step size, and an r^2^ threshold of 0.1. For admixture analyses, we modeled K (k = 2–5) ancestral populations with ADMIXURE and plotted the estimated proportion of ancestry of each sample using R. Three independent runs were performed for each value of K. The smallest cross validation error was obtained for K = 2 in this sample set, (mean of 0.365 versus mean CV-error of 0.368 for K = 3), however the results for K = 3 better correspond to the expected diversity of village and breed dogs.

## Additional files

Additional file 1: **Table S1.** SRA Sample IDs. (XLS 77 kb)

Additional file 2: **Table S2.** Summary of positions passing quality filters. (PDF 102 kb)

Additional file 3: **Figure S1.** Structure and annotation of the dog Y-chromosome (KP081776.1) amplicon region (chrY:1,200,000–2,440,580) A self-alignment of the dog Y-chromosome amplicon sequence for visualization of palindrome repeat sequences is presented (A). The three repeat families are color coded in purple, pink, and blue. The annotated sequence with amplicon genes (black) is represented as a UCSC browser track (B). Individual palindrome arms are numbered while palindrome spacers are lettered. (PDF 2240 kb)

Additional file 4: **Table S3.** Amplicon coordinates. (XLS 32 kb)

Additional file 5: **Figure S2.** Coyote read depth and MQ0 ratio by position An exponentially weighted moving averages (EWMA) of read depth (blue line) and the mq0/unfiltered depth ratio (pink line) are plotted along the Y-chromosome sequence for the coyote sample. (PNG 164 kb)

Additional file 6: **Figure S3:** Individual dog and wolf haplogroup topologies from RAxML tree A. HG1–3, B. HG6 C. HG23-HG8, D. HG27, E. HG9, F. Asian Wolves. Bootstrap support is indicated above branches. The names of wolves and dogs are colored in red and black, respectively. The placement of the three ancient dogs, CTC, HXH, and NGD are indicated as triangles above the most exterior node or leaf where diagnostic mutations indicated their membership. Most bootstrap values from HG1–3 are not shown due to space limitations. (PDF 334 kb)

Additional file 7: **Figure S4.** Autosomal admixture amongst breed dogs, village dogs and wolves. Barplots of ancestry proportions estimated by ADMIXTURE are shown for K values 2–5. Breed dogs, village dogs, and wolves are grouped and ordered from left to right. (PDF 1138 kb)

Additional file 8: Y-chromosome SNV genotypes. (GZ 35059 kb)

Additional file 9: **Table S4.** Diagnostic mutations for each major branch. (XLS 132 kb)

## Abbreviations

GATK: Genome Analysis Tool Kit
MCMC: Markov chain Monte Carlo
PCA: principal component analysis
SNP: single nucleotide polymorphism
SNV: single nucleotide variant
TMRCA: time to the most recent common ancestor
