# Supplementary material for: Analysis of the canid Y-chromosome phylogeny using short-read sequencing data reveals the presence of distinct haplogroups among Neolithic European dogs
Source: BMC Genomics. 2018 May 10;19:350. doi: 10.1186/s12864-018-4749-z (PMC5946424; doi:10.1186/s12864-018-4749-z)
Supplement: Supplementary file 2 — Table S2. Summary of positions passing quality filters. (PDF 102 kb) [file 12864_2018_4749_MOESM2_ESM.pdf]

Table S2 Summary of positions passing quality filters

**Filter**	**Passing Sites**	**Description**
Positions in Reference	2,460,580	unfiltered sequence
Regional Filtering	868,575	sites passing depth and MQ0 Ratio Filters
Site-Level Filtering	497,722	site-level filtering of heterozygote, MQ0 Ratio, and sites with missing genotypes
Second Depth	488,818	positions with extreme depth values
Indel Filter	484,924	+/- 5bp from indels
