# Supplementary material for: Analysis of the canid Y-chromosome phylogeny using short-read sequencing data reveals the presence of distinct haplogroups among Neolithic European dogs
Source: BMC Genomics. 2018 May 10;19:350. doi: 10.1186/s12864-018-4749-z (PMC5946424; doi:10.1186/s12864-018-4749-z)
Supplement: Supplementary file 3 — Figure S1. Structure and annotation of the dog Y-chromosome (KP081776.1) amplicon region (chrY:1,200,000–2,440,580) A self-alignment of the dog Y-chromosome amplicon sequence for visualization of palindrome repeat sequences is presented (A). The three repeat families are color coded in purple, pink, and blue. The annotated sequence with amplicon genes (black) is represented as a UCSC browser track (B). Individual palindrome arms are numbered while palindrome spacers are lettered. (PDF 2240 kb) [file 12864_2018_4749_MOESM3_ESM.pdf]

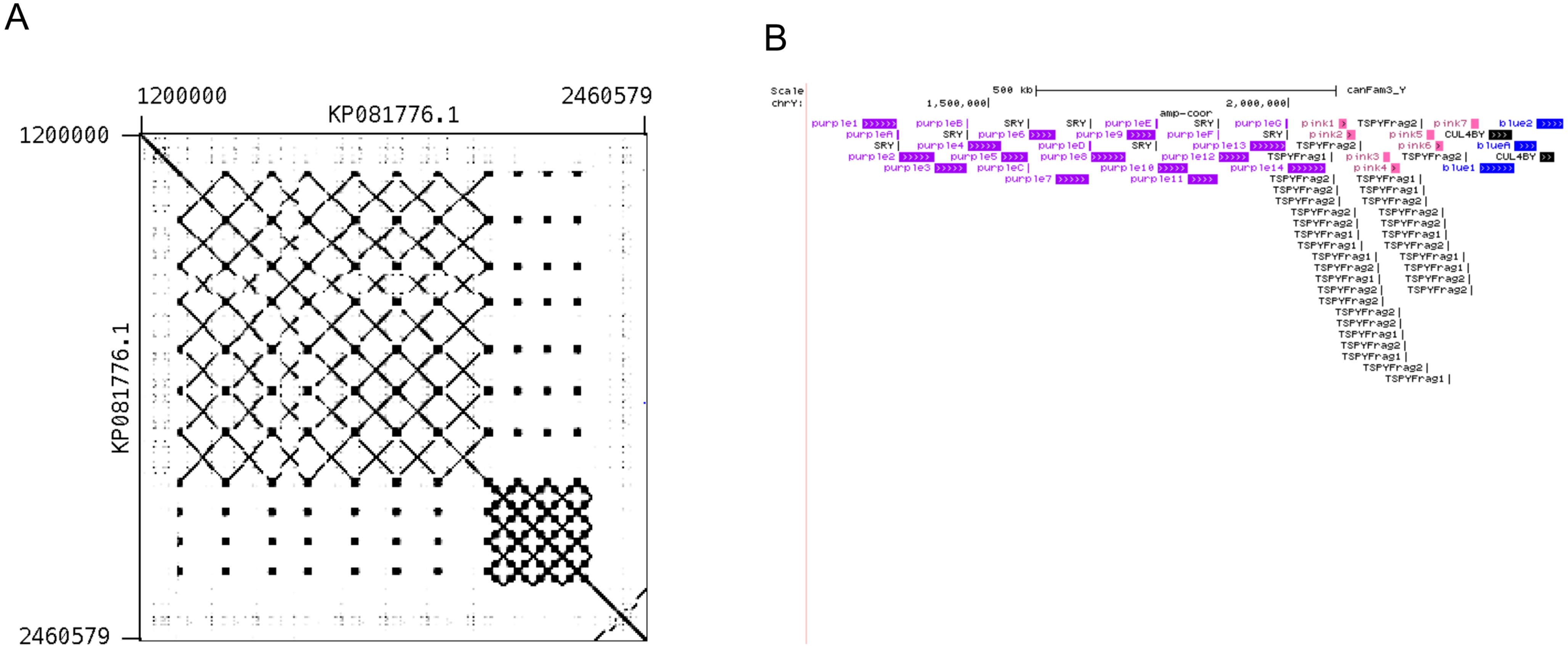
A
1200000
KP081776.1
2460579
1200000
KP081776.1
2460579
B
Scale
chrY:
500 kb
canFam3_Y
1,500,000
2,000,000
amp-coor
purple1
purpleB
SRY
purpleE
purpleG
pink1
TSPYFrag2
pink7
blue2
purpleA
purple6
purple9
purpleF
pink2
pink5
CUL4BY
purple4
purpleD
purple13
pink6
blueA
purple2
purple5
purple8
purple12
TSPYFrag1
pink3
purple3
purpleC
purple10
purple14
pink4
blue1
purple7
purple11
