# Supplementary figures and images for: Analysis of the canid Y-chromosome phylogeny using short-read sequencing data reveals the presence of distinct haplogroups among Neolithic European dogs
Source: BMC Genomics. 2018 May 10;19:350. doi: 10.1186/s12864-018-4749-z (PMC5946424; doi:10.1186/s12864-018-4749-z)

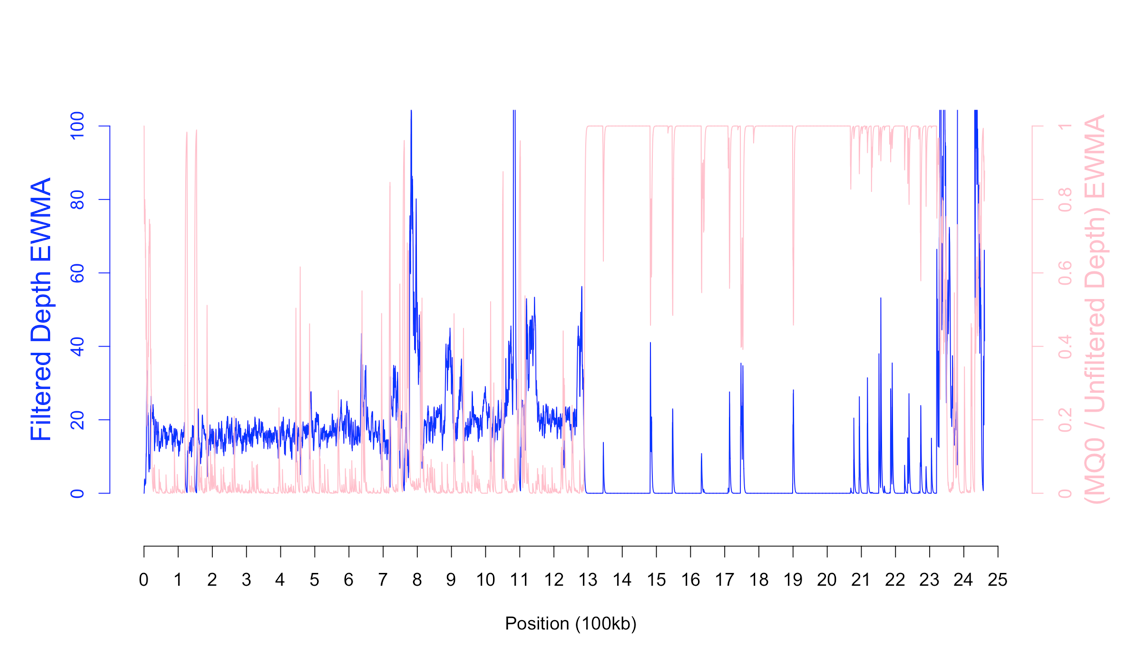

Supplement: Supplementary file 5 — Figure S2. Coyote read depth and MQ0 ratio by position An exponentially weighted moving averages (EWMA) of read depth (blue line) and the mq0/unfiltered depth ratio (pink line) are plotted along the Y-chromosome sequence for the coyote sample. (PNG 164 kb) [file 12864_2018_4749_MOESM5_ESM.png]

K=2

K=3

K=4

K=5

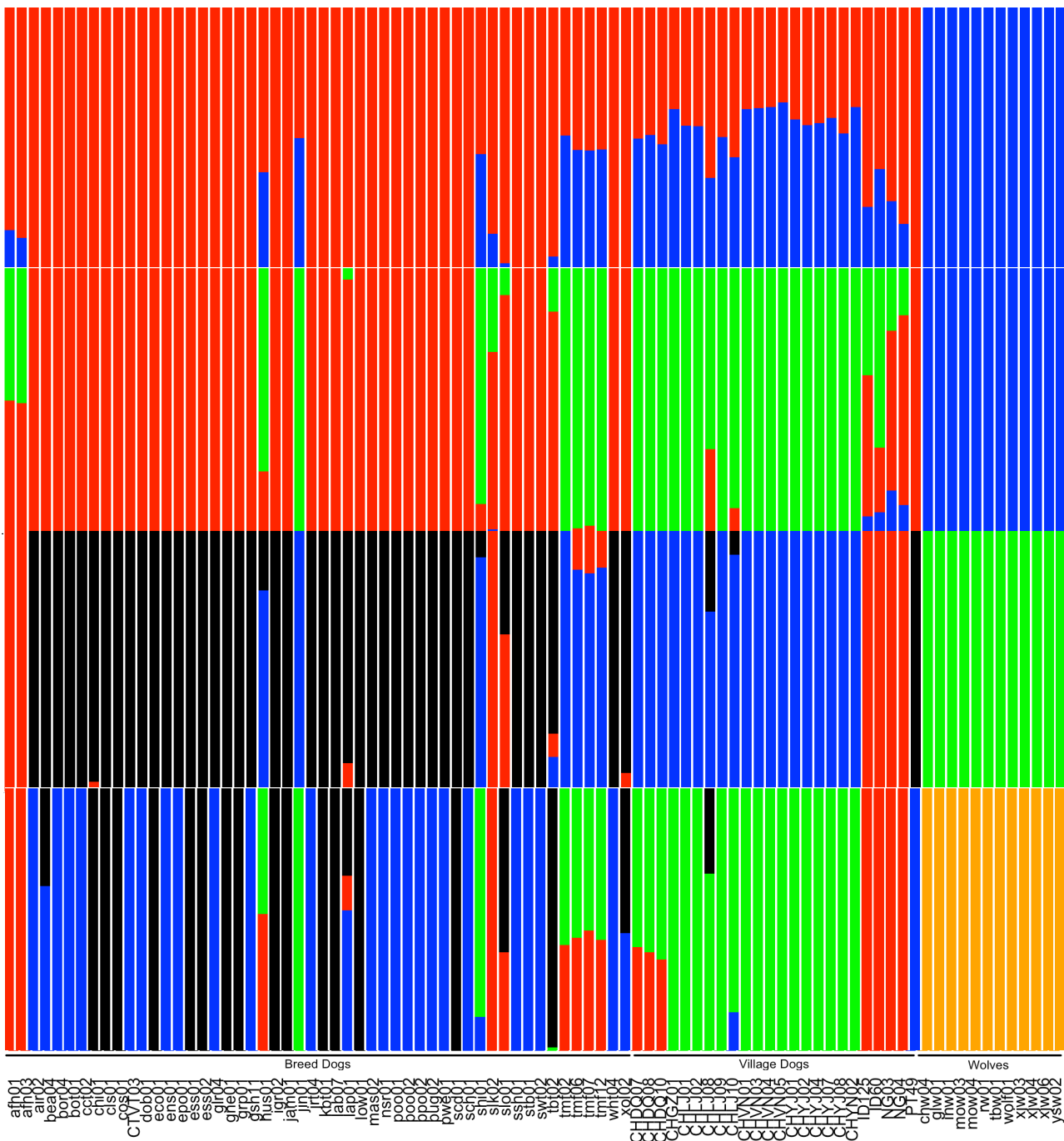

Supplement: Supplementary file 7 — Figure S4. Autosomal admixture amongst breed dogs, village dogs and wolves. Barplots of ancestry proportions estimated by ADMIXTURE are shown for K values 2–5. Breed dogs, village dogs, and wolves are grouped and ordered from left to right. (PDF 1138 kb) [file 12864_2018_4749_MOESM7_ESM.pdf]
