# Supplementary material for: Analysis of the canid Y-chromosome phylogeny using short-read sequencing data reveals the presence of distinct haplogroups among Neolithic European dogs
Source: BMC Genomics. 2018 May 10;19:350. doi: 10.1186/s12864-018-4749-z (PMC5946424; doi:10.1186/s12864-018-4749-z)
Supplement: Supplementary file 6 — Figure S3: Individual dog and wolf haplogroup topologies from RAxML tree A. HG1–3, B. HG6 C. HG23-HG8, D. HG27, E. HG9, F. Asian Wolves. Bootstrap support is indicated above branches. The names of wolves and dogs are colored in red and black, respectively. The placement of the three ancient dogs, CTC, HXH, and NGD are indicated as triangles above the most exterior node or leaf where diagnostic mutations indicated their membership. Most bootstrap values from HG1–3 are not shown due to space limitations. (PDF 334 kb) [file 12864_2018_4749_MOESM6_ESM.pdf]

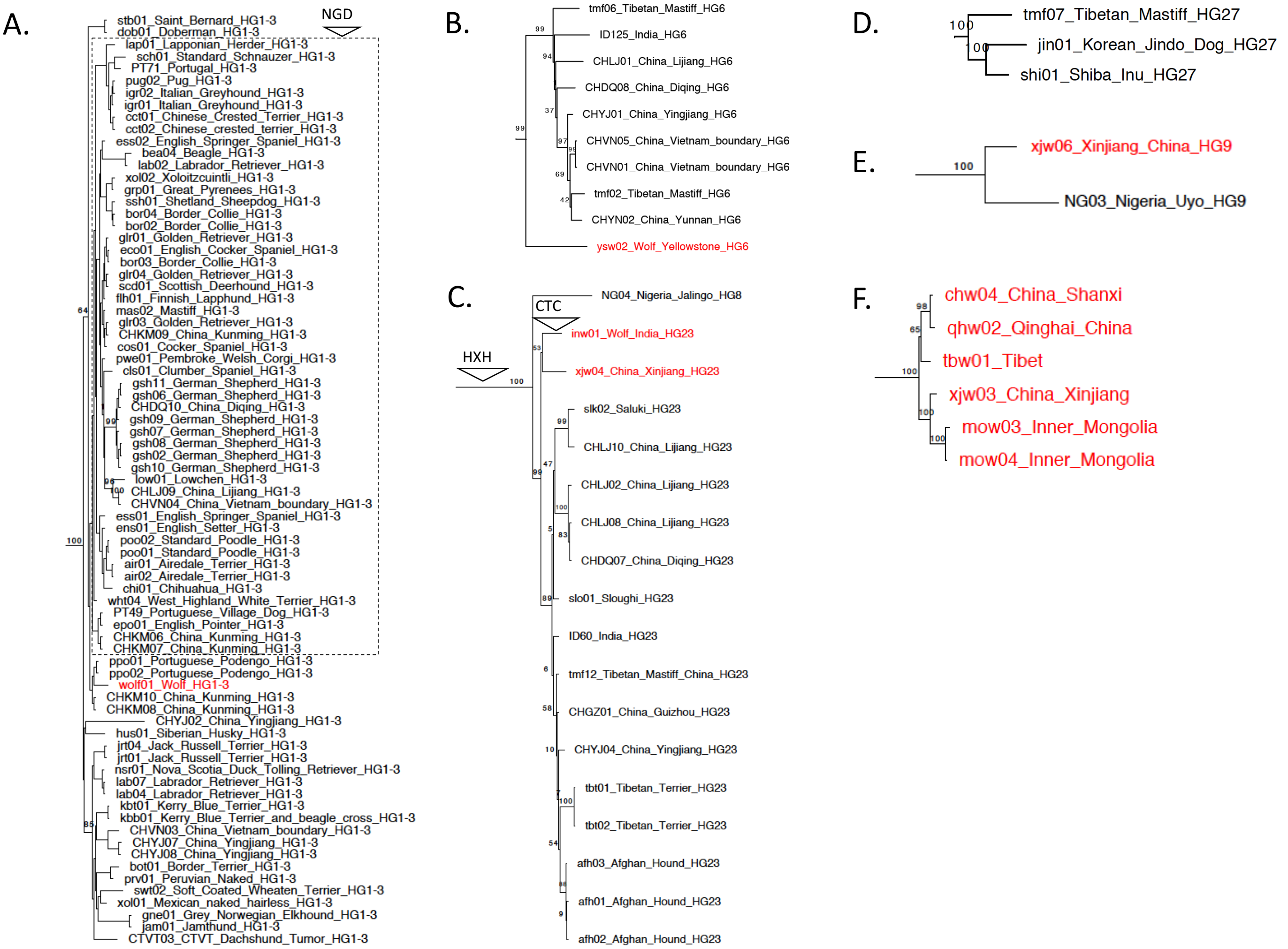

A.
NGD
stb01_Saint_Bernard_HG1-3
dob01_Doberman_HG1-3
lap01_Lapponian_Herder_HG1-3
sch01_Standard_Schnauzer_HG1-3
PT71_Portugal_HG1-3
pug02_Pug_HG1-3
igr02_Italian_Greyhound_HG1-3
igr01_Italian_Greyhound_HG1-3
cct01_Chinese_Crested_Terrier_HG1-3
cct02_Chinese_crested_terrier_HG1-3
ess02_English_Springer_Spaniel_HG1-3
bea04_Beagle_HG1-3
lab02_Labrador_Retriever_HG1-3
xol02_Xoloitzcuintli_HG1-3
grp01_Great_Pyrenees_HG1-3
ssh01_Shetland_Sheepdog_HG1-3
bor04_Border_Collie_HG1-3
bor02_Border_Collie_HG1-3
glr01_Golden_Retriever_HG1-3
eco01_English_Cocker_Spaniel_HG1-3
bor03_Border_Collie_HG1-3
glr04_Golden_Retriever_HG1-3
scd01_Scottish_Deerhound_HG1-3
flh01_Finnish_Lapphund_HG1-3
mas02_Mastiff_HG1-3
glr03_Golden_Retriever_HG1-3
CHKM09_China_Kunming_HG1-3
cos01_Cocker_Spaniel_HG1-3
pwe01_Pembroke_Welsh_Corgi_HG1-3
cls01_Clumber_Spaniel_HG1-3
gsh11_German_Shepherd_HG1-3
gsh06_German_Shepherd_HG1-3
CHDQ10_China_Diqing_HG1-3
gsh09_German_Shepherd_HG1-3
gsh07_German_Shepherd_HG1-3
gsh08_German_Shepherd_HG1-3
gsh02_German_Shepherd_HG1-3
gsh10_German_Shepherd_HG1-3
low01_Lowchen_HG1-3
CHLJ09_China_Lijiang_HG1-3
CHVN04_China_Vietnam_boundary_HG1-3
ess01_English_Springer_Spaniel_HG1-3
ens01_English_Setter_HG1-3
poo02_Standard_Poodle_HG1-3
poo01_Standard_Poodle_HG1-3
air01_Airedale_Terrier_HG1-3
air02_Airedale_Terrier_HG1-3
chi01_Chihuahua_HG1-3
wht04_West_Highland_White_Terrier_HG1-3
PT49_Portuguese_Village_Dog_HG1-3
epo01_English_Pointer_HG1-3
CHKM06_China_Kunming_HG1-3
CHKM07_China_Kunming_HG1-3
ppo01_Portuguese_Podengo_HG1-3
ppo02_Portuguese_Podengo_HG1-3
wolf01_Wolf_HG1-3
CHKM10_China_Kunming_HG1-3
CHKM08_China_Kunming_HG1-3
CHYJ02_China_Yingjiang_HG1-3
hus01_Siberian_Husky_HG1-3
jrt04_Jack_Russell_Terrier_HG1-3
jrt01_Jack_Russell_Terrier_HG1-3
nsr01_Nova_Scotia_Duck_Tolling_Retriever_HG1-3
lab07_Labrador_Retriever_HG1-3
lab04_Labrador_Retriever_HG1-3
kbt01_Kerry_Blue_Terrier_HG1-3
kbb01_Kerry_Blue_Terrier_and_beagle_cross_HG1-3
CHVN03_China_Vietnam_boundary_HG1-3
CHYJ07_China_Yingjiang_HG1-3
CHYJ08_China_Yingjiang_HG1-3
bot01_Border_Terrier_HG1-3
prv01_Peruvian_Naked_HG1-3
swt02_Soft_Coated_Wheaten_Terrier_HG1-3
xol01_Mexican_naked_hairless_HG1-3
gne01_Grey_Norwegian_Elkhound_HG1-3
jam01_Jamthund_HG1-3
CTVT03_CTVT_Dachshund_Tumor_HG1-3
B.
tmf06_Tibetan_Mastiff_HG6
ID125_India_HG6
CHLJ01_China_Lijiang_HG6
CHDQ08_China_Diqing_HG6
CHYJ01_China_Yingjiang_HG6
CHVN05_China_Vietnam_boundary_HG6
CHVN01_China_Vietnam_boundary_HG6
tmf02_Tibetan_Mastiff_HG6
CHYN02_China_Yunnan_HG6
ysw02_Wolf_Yellowstone_HG6
C.
HXH
CTC
NG04_Nigeria_Jalingo_HG8
inw01_Wolf_India_HG23
xjw04_China_Xinjiang_HG23
slk02_Saluki_HG23
CHLJ10_China_Lijiang_HG23
CHLJ02_China_Lijiang_HG23
CHLJ08_China_Lijiang_HG23
CHDQ07_China_Diqing_HG23
slo01_Sloughi_HG23
ID60_India_HG23
tmf12_Tibetan_Mastiff_China_HG23
CHGZ01_China_Guizhou_HG23
CHYJ04_China_Yingjiang_HG23
tbt01_Tibetan_Terrier_HG23
tbt02_Tibetan_Terrier_HG23
afh03_Afghan_Hound_HG23
afh01_Afghan_Hound_HG23
afh02_Afghan_Hound_HG23
D.
tmf07_Tibetan_Mastiff_HG27
jin01_Korean_Jindo_Dog_HG27
shi01_Shiba_Inu_HG27
E.
xjw06_Xinjiang_China_HG9
NG03_Nigeria_Uyo_HG9
F.
chw04_China_Shanxi
qhw02_Qinghai_China
tbw01_Tibet
xjw03_China_Xinjiang
mow03_Inner_Mongolia
mow04_Inner_Mongolia
